# Qualitative and Quantitative Assessments of Apple Quality Using Vis Spectroscopy Combined with Improved Particle-Swarm-Optimized Neural Networks

**DOI:** 10.3390/foods12101991

**Published:** 2023-05-15

**Authors:** Wenping Peng, Zhong Ren, Junli Wu, Chengxin Xiong, Longjuan Liu, Bingheng Sun, Gaoqiang Liang, Mingbin Zhou

**Affiliations:** 1 Key Laboratory of Optic-Electronics and Communication, Jiangxi Science and Technology Normal University, Nanchang 330038, China; 2 Key Laboratory of Optic-Electronic Detection and Information Processing of Nanchang City, Jiangxi Science and Technology Normal University, Nanchang 330038, China

**Keywords:** quality assessment, soluble solid content, Vis spectra, dynamic learning rate nonlinear decay strategy, particle swarm optimization

## Abstract

Exploring a cost-effective and high-accuracy optical detection method is of great significance in promoting fruit quality evaluation and grading sales. Apples are one of the most widely economic fruits, and a qualitative and quantitative assessment of apple quality based on soluble solid content (SSC) was investigated via visible (Vis) spectroscopy in this study. Six pretreatment methods and principal component analysis (PCA) were utilized to enhance the collected spectra. The qualitative assessment of apple SSC was performed using a back-propagation neural network (BPNN) combined with second-order derivative (SD) and Savitzky–Golay (SG) smoothing. The SD-SG-PCA-BPNN model’s classification accuracy was 87.88%. To improve accuracy and convergence speed, a dynamic learning rate nonlinear decay (DLRND) strategy was coupled with the model. After that, particle swarm optimization (PSO) was employed to optimize the model. The classification accuracy was 100% for testing apples via the SD-SG-PCA-PSO-BPNN model combined with a Gaussian DLRND strategy. Then, quantitative assessments of apple SSC values were performed. The correlation coefficient (*r*) and root-square-mean error for prediction (RMSEP) in testing apples were 0.998 and 0.112 °Brix, surpassing a commercial fructose meter. The results demonstrate that Vis spectroscopy combined with the proposed synthetic model has significant value in qualitative and quantitative assessments of apple quality.

## 1. Introduction

Apples, as one of the most nutritious fruits, are loved by consumers around the world because of their rich vitamin content and trace elements. Apples are also one of the most widely profitable fruits, accounting for 15% of all fruit sales worldwide [1]. At present, more than 80 countries and regions produce apples around the world, with the main production areas being concentrated in Asia, Europe, and North America. Asia ranks first and Europe ranks second in the world. In 2022, the world’s apple production reached 81.799 million tons; China and the European Union are the major regions of global apple consumption, with consumption accounting for 54% and 14%, respectively. It is expected that global apple production will reach 100 million tons by 2025. The quality of apples varies between regions due to environmental factors, soil characteristics, etc. Most traditional methods of assessing apple quality are based on observing color, size, and taste. These methods have interference problems associated with subjective factors, which result in inaccurate assessments and low efficiency. Chemical identification methods are usually time-consuming and unsuitable for mass operations. There are also few studies on qualitative and quantitative assessments of apple quality. For example, Zheng et al. [2] used two-dimensional electrophoresis and mass spectrometry analysis to qualitatively and quantitatively evaluate the quality of apples and strawberries. However, the evaluation’s efficiency was limited by the complexity of extracting the protein of fruit tissues and the low performance-to-price ratio of the detection instruments, which is indicative of the great difficulty in sorting apples onsite or online. Therefore, developing a convenient and cost-effective method of qualitatively and quantitatively assessing apple quality would be of significant value. 

Over the last decade, spectroscopy technology [3] has become a research hotspot in achieving better fruit quality detection. This technology can rapidly detect fruit quality because of the advantages of a short detection time, simple operation, and the simultaneous detection of multiple components. To date, it has been widely used for sugar content, regional traceability, and even identifying fruit varieties, such as apples and pears (Mo et al. [4]; Huang et al. [5]; Shen et al. [6]; Bat et al. [7]; Mendoza et al. [8]). In addition, it has some applications in fruit quality detection; for example, Song et al. [9] proposed a method using near-infrared spectroscopy (NIRS) combined with pattern recognition, in which the spectra were preprocessed via baseline correction and normalization. A partial least-squares discriminant analysis (PLS-DA) model was used for classification, and apple samples were classified as organic or non-organic with an accuracy of 96%. Tran et al. [10] combined an optimally trained classifier with a self-developed simple spectroscopy system to classify the sweetness of apples, and a classification accuracy of 91.3% was obtained using a discriminant analysis (DA) model. Zhang et al. [11] employed NIRS to detect apple ripeness via iodine testing, classifying apples into three categories of ripeness (i.e., immaturity, harvest maturity, and edible maturity), and a linear DA model was developed by using spectral indices. The classification accuracy of the testing-set samples was 87.15%. In addition, the classification accuracies of the testing-set samples were 87.36% and 90.11%, respectively, for the least-square method coupled with a support vector machine (LS-SVM) model using raw spectra or combined with a successive projection algorithm (SPA) to extract 11 features. These results show that NIRS techniques have potential value and feasibility in assessing fruit quality. 

There have also been studies on fruit quality using visible and near-infrared spectroscopy (Vis/NIRS). For example, Xu [12] utilized Vis/NIRS to detect the internal flavors of the ‘Shatian’ pomelo fruit. The coefficient of determination and the root-mean-square error (RMSE) of the validation set for soluble solid content (SSC) were 0.72 and 0.49 °Brix, respectively, and for acidity detection, the values were 0.55% and 0.10%, respectively. Hua [13] used visible/shortwave–near-infrared spectroscopy (Vis/SW-NIRS) with light-emitting diode light sources and a silicon photodetector to measure the SSC and firmness of wax apples. Partial least squares regression (PLSR) was used to build calibration modes and analyze the prediction of the correlation (*r*_p_^2^) and the root-mean-square error for prediction (RMSEP). The results showed that the *r*_p_^2^ and RMSEP values were 0.87 and 0.66 °Brix, respectively, in SSC measurements and 0.80 and 1.16 N/cm^2^, respectively, in firmness measurements. Fan [14] employed Vis/NIRS with diffuse transmittance to determine the SSC of “Fuji” apples at 650–910 nm. Partial least squares (PLS) models were combined with a modified competitive adaptive reweighted sampling (MCARS), and SPA was used to analyze the performance of the SSC determination. The correlation coefficients between the measured and predicted SSC were 0.962 and 0.946, and the RMSE values were 0.510 °Brix and 0.527 °Brix for the calibration and prediction sets, respectively. Torres [15] used Vis/NIRS combined with chemometric techniques to identify bitter pits in Golden apples, as well three different classification algorithms, i.e., linear discriminant analysis (LDA), quadratic discriminant analysis (QDA), and the support vector machine (SVM). The detection accuracies for pitted apples with visible symptoms postharvest were 75–81%. Scalisi [16] studied the application of Vis/NIRS in estimating SSC, dry matter concentration (DMC), and flesh firmness in nectarines, peaches, apricots, and Japanese plums at the time of harvest by using PLSR models based on the second derivative (SD) of the absorbance occurring in the 729–975 nm spectral region; the accuracy (*R*^2^_CV_) of the SSC and DMC predictions was larger than 0.75.

Although there are advantages to using NIRS for spectral detection, NIRS instruments are usually expensive, especially desktop Fourier transform NIR spectrometers and grating spectrometers with higher optical resolutions. This results in a low performance-to-cost ratio. At the same time, although some studies on detecting fruit quality based on Vis/NIRS have already been conducted, there are few works that have studied fruit quality detection and assessment based on Vis spectroscopy alone. Therefore, it is necessary to seek a highly cost-effective spectral detection technology to qualitatively and quantitatively assess fruit quality. Studies report that some visible wavelengths, e.g., 461 nm, 469 nm [17], and 750 nm, are the characteristic absorption wavelengths of the carbohydrates in fruits, such as SSC and proteins. It has been demonstrated that spectral-detection-based Vis spectroscopy is feasible. Of note, the performance-to-cost ratio of spectral-detection-based Vis spectroscopy is higher than that of NIRS. 

As with NIRS spectra, Vis spectra generally contain a large amount of information and require multivariate statistical techniques for analysis. Artificial neural networks (ANN) are powerful analytical tools for qualitative classification and quantitative prediction. They have the advantages of high accuracy in classification, strong parallel distributed processing, fast learning speeds, and strong resistance to noisy nerves and fault tolerance [18]. These advantages make applying ANN to fruit quality detection possible. For example, Opeña et al. [19] developed an automatic tomato classification system using an ANN classifier trained with the artificial bee colony (ABC) algorithm, i.e., the ABC-trained ANN classifier, which performed tomato classification with an accuracy of 98.19%.

**Figure 1:**
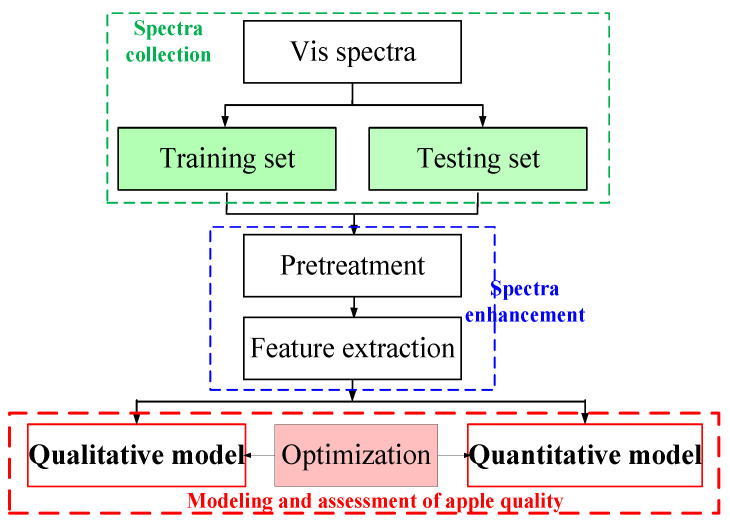
Basic framework of qualitative and quantitative assessment of apple quality.

Our main aim is to develop a highly accurate and high performance-to-cost ratio assessment scheme to detect apple quality via Vis spectroscopy; to establish qualitative and quantitative models via ANN; and, finally, to ensure the high classification accuracy and sufficiently low RMSE value of apple SSC values by using spectra enhancement and intelligent optimization methods. In this work, SSC was used to evaluate the quality of apples. SSC normally refers to the percentage concentration of soluble sugars (mainly fructose, glucose, and sucrose) in fruit, and there is a significant positive correlation between SSC and soluble sugars [20]. SSC is an important apple quality element because of its commercial value and intuitive taste. Therefore, SSC is commonly used in production to compare the sugar contents of fruit. In general, apples with a high and uniform SSC (≥13 °Brix) have a sweeter and more delicious taste, which is preferred by consumers [4]. The basic framework of qualitative and quantitative assessments of apple SSC values using Vis spectroscopy is provided in Figure 1.

The contents of this study are as follows: (1) The Vis absorption spectra of apples were collected using a UV-Vis spectrometer in transmission mode, and the morphological differences in the spectra of apples with different SSC values were analyzed. At the same time, the actual SSC values of the apples were measured using a commercial refractive fructose meter. (2) To improve the assessment accuracy, six different pretreatment methods were performed, and principal component analysis (PCA) was used to extract the main feature information of the raw Vis spectra of apples. (3) To qualitatively and quantitatively assess apple quality based on SSC, a qualitative classification model of SSC based on the back-propagation neural network (BPNN) model was established for the pretreated and data-augmented spectra. Moreover, four dynamic learning rate nonlinear decay (DLRND) strategies combined with BPNN were used to improve the classification accuracy and increase the convergence speed. (4) To further improve the classification accuracy, the particle swarm optimization (PSO) algorithm was used to optimize the BPNN model. To validate the availability of the synthetic method proposed in this work, i.e., the second-order derivative + Savitzky–Golay(SG) smoothing + PCA + PSO + BPNN (SD-SG-PCA-PSO-BPNN) model, we combined it with a Gaussian DLRND strategy; the qualitative classification accuracy of apple SSC based on this synthetic method was compared with that of several other models. (5) Based on the proposed synthetic method, the quantitative assessment of apple SSC was also studied. The availability of quantitatively predicted apple SSC based on this synthetic method was validated using RMSE values and correlation coefficients between several quantitative assessment algorithms.

## 2. Materials and Methods

### 2.1. Sample Preparation

**Figure 2:**
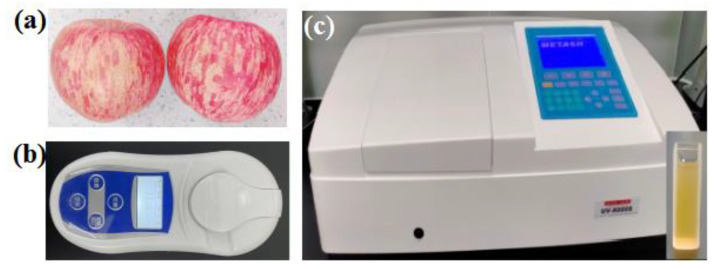
Apple samples (**a**), the commercial refractive fructose meter (**b**), and UV-Vis spectrophotometer and cuvette (**c**).

In the experiments, 100 red Fuji apples were purchased from local fruit markets in Luochuan, Shanxi, China; the representative apples are shown in Figure 2a. All of these apples were free from bruising or decay and were visually similar in size. Prior to measurement, all apples were packed in sealed plastic bags and stored in a refrigerator (4 ± 0.5 °C). Before the experiments, these apples were taken out of the refrigerator and washed with water. To avoid interference from exogenous water, the remaining water on the surface of the apples was wiped off with absorbent paper; then, they were placed on pallets and left to naturally airdry for 24 h. After that, the pulp juice of each apple was extracted using a manual fruit juicer at room temperature (20 ± 0.5 °C). The extracted juice of each apple was filtered by using the filter nets with 200 meshes.

### 2.2. SSC Measurement

Approximately 1.0 mL of filtered juice was removed to measure the actual SSC level of each apple via a commercial refractive fructose meter (PAL-107, Qiwei Instrument Co., Ltd., Hangzhou, China) with a range of 0–53 °Brix and an accuracy deviation of ±0.2 °Brix, as shown in Figure 2b. Each sample was measured three times, and then, the average value was used for data analysis. 

### 2.3. Statistical Analysis

In this work, 100 apple samples were categorized into 3 different grades according to the SSC level. In Grade 1, the top grade, the SSC range is from 14 to 17.99 °Brix. In Grade 2, the medium grade, the SSC range is from 10 to 13.99 °Brix. In Grade 3, the poor grade, the SSC range is from 8 to 9.99 °Brix. The statistical results for the SSC values of all the apple samples based on analysis of variance (ANOVA) were obtained using the SPSS 20.0 statistics software (IBM SPSS Co., Chicago, IL, USA).

### 2.4. Spectral Acquisition

The absorbance spectra of apple samples were collected by using a UV-Vis spectrophotometer (UV8000S, Metash Co., Shanghai, China), as shown in Figure 2c. The UV-Vis spectrophotometer is a dual-beam spectrophotometer with the wavelength range of 190 nm to 1100 nm with wavelength accuracy of ±0.3 nm and photometric accuracy of ±0.002 Abs (0–0.5 Abs). A quartz cuvette with an optical path of 10 mm and volume of 3.5 mL was used to load apple juice, as shown in Figure 2c. All measurements were performed at room temperature (20 ± 0.5 °C).

### 2.5. Spectral Pretreatment

The raw spectra of the apple samples may contain noise and other useless information, and spectra with low signal-to-noise ratios will seriously impact the availability of data modeling and the accuracy of qualitative and quantitative SSC assessment. Therefore, it is necessary to perform spectral pretreatment on the raw spectra of apple samples. Some pretreatment methods are commonly used in spectral analysis, such as mean centering [21], data standardization [22], vector normalization (VN) [23], Savitzky–Golay (SG) smoothing [24], first-order derivative (FD) [25] or second-order derivative (SD) [26] methods, multiplicative scatter correction (MSC) [27], and standard normal variate (SNV) transformation [28]. In practice, several pretreatment methods are sometimes combined to obtain satisfactory results. This study investigated and compared the effects of six spectral pretreatment methods, i.e., FD, SD, SNVT, VN, SG smoothing, and SD-SG, on the qualitative classification accuracy of models for apple SSC. 

### 2.6. Spectral Features Extraction

In a full spectrum, extracting the valuable characteristic wavelengths not only reduces the number of variables, i.e., data dimensionality reduction, but also makes the model simpler and more robust and improves its interpretability. This study employed a principal component analysis (PCA) algorithm [29] to extract the main spectral features of SSC from six pretreatment apple spectra. In general, the number of suitable latent variables (LVs), i.e., principal components (PCs), is determined based on the cumulative contribution rate of the first *k* LVs with large contribution rates, which needed to be greater than or equal to 95% in this work. The contribution rate of the *i*th LVs and the cumulative contribution rate of *k* LVs are calculated according to Equations (1) and (2):

(1) $$\varphi_{i}=\frac{\lambda_{i}}{\sum_{1}^{m}\lambda_{i}}$$

(2) $$\phi \left(k\right)=\sum_{i=1}^{k}\varphi_{i}$$

where *λ_i_* is the eigenvalue of *i*th LVs, *m* is the total dimensionality of the spectra, $\varphi_{i}$ is the *i*th variance contribution rate, and *k* is the number of LVs.

### 2.7. Qualitative and Quantitative Assessments Modeling

**Figure 3:**
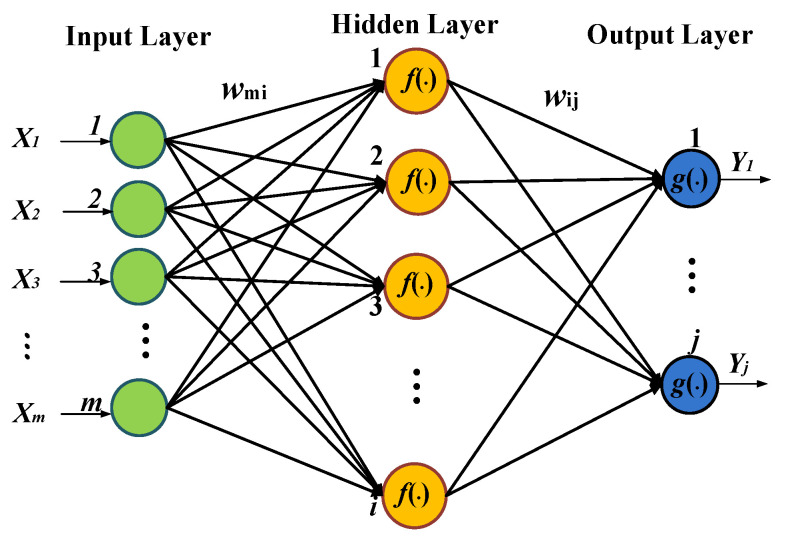
Structure of three-layer BPNN model.

In this work, qualitative classifications and quantitative assessments of apple SSC were studied using an ANN model, i.e., a back-propagation neural network (BPNN) [30,31]. Figure 3 shows the structure of the three-layer BPNN model.

Combined with six pretreatment methods, the LV scores of the apple Vis spectra extracted using PCA were used as input data for the BPNN, and the number of neurons in the output layer was set to 3, corresponding to the 3 SSC level grades. The number of neurons in the hidden layer was determined according to the following empirical formula [32]:

(3) $$h=\sqrt{m+n}+a$$

where *h* is the number of neurons in the hidden layer; *m* and *n* are the numbers of neurons in the input layer and output layer, respectively; and *a* is an adjustable constant ranging between 1 and 10.

In the BPNN model, to avoid unbalanced distribution in the three apple sample grades in the original dataset and improve the robustness and performance of the classifier, data augmentation based on the synthetic minority over-sampling technique (SMOTE) algorithm [33] was performed on the original dataset. That is, we increased the number of Grade 1 and Grade 3 apple samples by interpolating a few sample classes, though the number of Grade 2 apple samples remained the same. Ultimately, the augmented dataset contained 165 samples, of which there were 66 samples in Grade 1, 71 in Grade 2, and 28 in Grade 3. The calibration set (or training set) and the prediction set (or testing set) were randomly divided with a ratio of 8:2 based on the stratified random sampling method [34] and the hold-out method [35]. Then, the training-set samples were used to establish the assessment models, and the classification accuracy of the testing samples was used as the evaluation indicator. Data processing and modeling apple SSC values were performed using the programming software Matlab 2021b (MathWorks, Inc., Natick, MA, USA).

### 2.8. Dynamic Learning Rate Decay Strategy of BPNN Modeling

For the BPNN model, the gradient descent method was applied to the neural network. In the gradient descent method, a key controlling factor, i.e., the learning rate, *η*, is one of the hyperparameters that affects the optimization performance [36]. Although the learning rate is often set as a constant in BPNNs, there are problems in practice; that is, a too-large learning rate will result in too-large weight updating, and the model’s performance will be unstable during the training process. Conversely, a too-small learning rate may never converge or fall into a local optimum solution. To solve these problems for the constant learning rate, the dynamic learning rate nonlinear decay (DLRND) method was employed in this work. That is, a larger learning rate was used for model optimization at the early stage of training, and then, the learning rate was gradually reduced with an increase in the iteration times to ensure the model did not fluctuate too much in the later stages of training and to keep convergence optimal [37]. 

In this work, four DLRND strategies (i.e., exponential decay [38], cosine decay [39], Gaussian decay [40], and Sigmoid decay [41]) were used and compared, which are presented as follows:

(4) $$\left\{\begin{array}{c} \eta_{\exp}\left(t\right)=\eta_{0}{\left(1-\frac{t}{T}\right)}^{p}\\ \eta_{\cos}\left(t\right)=0.5\times \eta_{0}\left(1+\cos\left(\frac{t\pi }{T}\right)\right)\\ \eta_{\mathrm{Gaus}}\left(t\right)=\eta_{0}e^{-\frac{t^{2}}{2\cdot \gamma^{2}}}\\ \eta_{\mathrm{Sigm}}\left(t\right)=\eta_{0}{\left(1+e^{-\gamma \left(t-\frac{T}{2}\right)}\right)}^{-1} \end{array}\right.$$

where *η*_exp_(*t*), *η*_cos_(*t*), *η*_Gaus_(*t*), and *η*_Sigm_(*t*) are the exponential decay strategy, cosine decay strategy, Gaussian decay strategy, and Sigmoid decay strategy, respectively. *η*_0_ is the initial learning rate, *p* is the coefficient (*p* > 1: concave curve; 0 < *p* < 1: convex curve), and *T* is the total number of iterations. γ is the attenuation rate.

**Figure 4:**
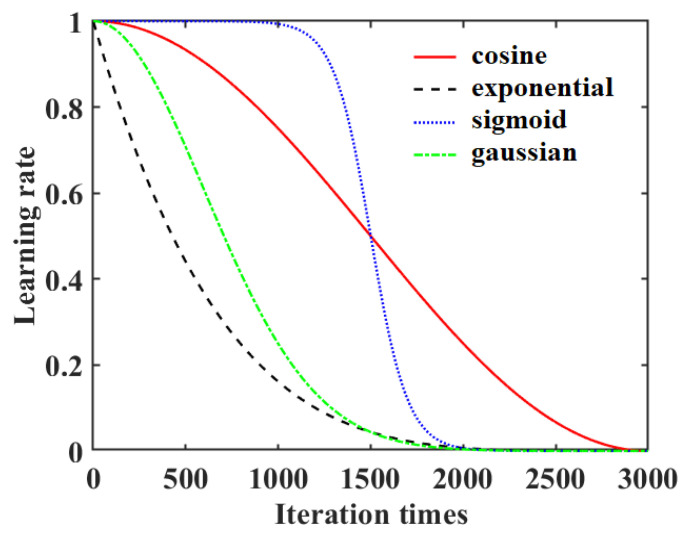
Comparison of four different DLRND strategies.

Figure 4 shows the profiles of different DLRND strategies (assuming an initial learning rate of 1).

### 2.9. Particle Swarm Optimization (PSO)-Optimized BPNN Algorithm

Although the BPNN algorithm is a gradient-descent-based method and has the advantages of nonlinear mapping, self-learning, and adaptive capability, it also has some disadvantages, such as slow convergence, and it easily falls into the local optimum. To further improve the classification accuracy of apple SSC, the particle swarm optimization (PSO) algorithm [42,43] was applied to the SD-SG-PCA-BPNN model, combined with the Gaussian DLRND strategy, to optimize the weights and thresholds of the BPNN.

For PSO, each particle contains two properties, i.e., the velocity vector (*v*) and the position vector (*x*). All particles in the swarm adjust their velocities and positions according to the current individual and population extremes. The particles should update their velocities and positions based on the following equations:

(5) $$v_{i}^{\left(k+1\right)}=\omega \times v_{i}^{\left(k\right)}+c_{1}\times rand()\times \left(pbest_{i}^{\left(k+1\right)}-x_{i}^{\left(k\right)}\right)+c_{2}\times rand()\times \left(gbest_{i}^{\left(k+1\right)}-x_{i}^{\left(k\right)}\right)$$

(6) $$x_{i}^{\left(k+1\right)}=x_{i}^{\left(k\right)}+v_{i}^{\left(k+1\right)}$$

where the subscript *i* = 1, 2, ..., *N*, *N* is the total number of particles in the population, *ω* is the inertia weight factor, $v_{i}^{\left(k\right)}$ is the velocity of the *i*th particle at moment *k*, rand() is a random number between 0 and 1, $pbest_{i}^{\left(k+1\right)}$ and $gbest_{i}^{(k+1)\;}$ are the individual optimum and the global optimum, respectively, $v_{i}^{\left(k\right)}$ is the current position of the *i*th particle at moment *k*, and *c*_1_ and *c*_2_ are two acceleration factors.

## 3. Results and Analysis

### 3.1. Statistical Analysis of SSC

**Figure 5:**
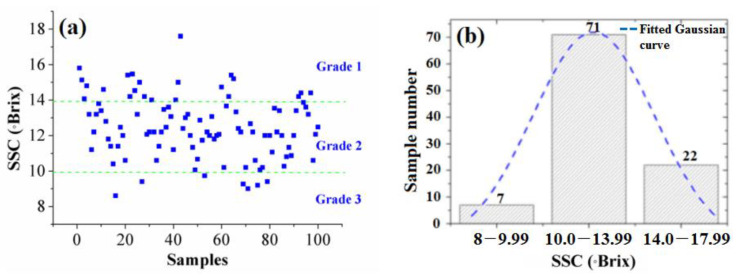
SSC values (**a**) and number distributions for three grades of apples (**b**).

The SSC values of 100 apple samples are presented in Figure 5a. One hundred apple samples were categorized into three different grades (i.e., Grade 1, Grade 2, and Grade 3) according to the SSC level. Based on the analysis of variance (ANOVA), the statistical analysis results of the SSC values for each apple sample grade are shown in Table 1. *p* < 0.05 was considered significant. 

The number of Grade 1, Grade 2, and Grade 3 apples was 22, 71, and 7, respectively. The number distributions for the three grades are shown in Figure 5b.

Figure 5a,b show that the number of apples in the medium grade is the largest, and the number of apples in the top grade and poor grade is smaller. Figure 3 shows the number distribution of the apples conforming to the Gaussian function, which demonstrates that our choice of apple samples was satisfactory. 

### 3.2. Spectral Acquisition

**Figure 6:**
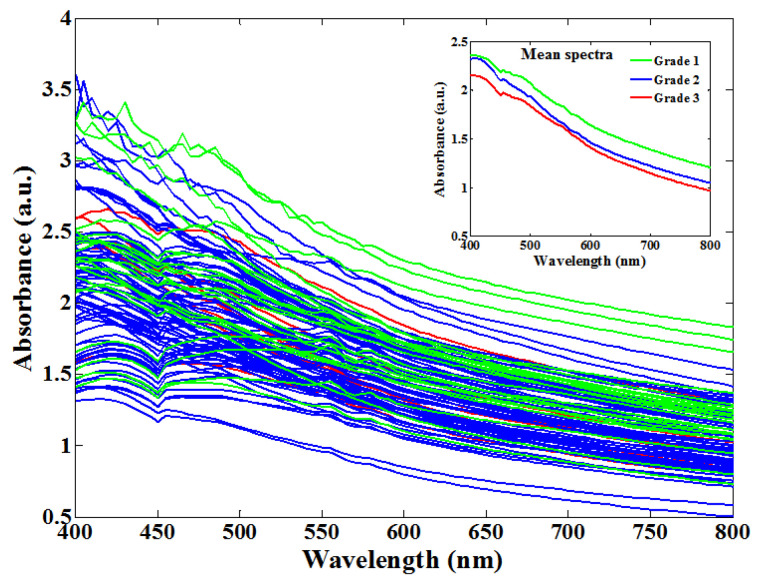
Raw Vis spectra and mean spectra of apples in 400–800 nm.

In this study, the absorption spectra of 100 apple samples were collected in a wavelength of 400–800 nm with an interval of 5 nm, as shown in Figure 6. Although the profiles of all the apples’ absorption spectra looked similar, the absorbance of each apple was different, which might be due to the different SSC levels of the apples. That is, the Vis spectra of the apples have unique fingerprint effects, which can be used to qualitatively and quantitatively assess the apple quality via data processing and modeling methods. 

Figure 6 shows that the whole-spectra profiles of all the apples look similar. Although the spectra of apples with different SSC values overlap, the mean spectra of all three degrees of SSC can be distinguished, which are presented in the subgraph of Figure 6. From the mean spectra of the apples, we can see that the absorbance is, to an extent, proportional to the SSC of the apples in a fixed optical path, which is consistent with the Lambert–Beer law [44]. Therefore, the SSC value of an apple can be determined based on its spectral absorbance using quantitative modeling methods. At the same time, a quality assessment of apples can also be achieved.

### 3.3. Spectral Pretreatment

**Figure 7:**
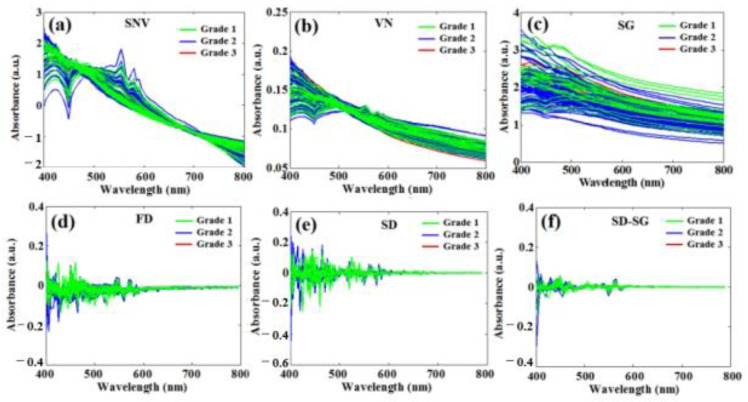
Pretreated spectra of apples based on six kinds of pretreatment methods. (**a**) SNV; (**b**) VN; (**c**) SG smoothing; (**d**) FD; (**e**) SD; (**f**) SD-SG smoothing.

The pretreated spectra of 100 apple samples obtained by using six pretreatment methods, i.e., SNV, VN, SG smoothing, FD, SD, and SD-SG, are shown in Figure 7a–f, respectively.

From Figure 7a,b, although the principles behind the SNV and VN pretreatments are different, the pretreated spectra are also basically similar because of their approximate mathematical form. By using the SNV and VN pretreatment methods, the differences between different grades of apple samples are significantly reduced, and the differences between the spectra of apple samples in the same grade are also reduced. At the same time, the influence of the inhomogeneity of apple juice filtered through filter gauze can be reduced by using SNV and VN. Figure 7c shows that the SG smoothing pretreatment does not change the profiles of the spectra, but the noises can be significantly reduced. Figure 7d shows that the FD pretreatment method can separate the overlapping absorption peaks from the raw spectra, remove the spectra bias and baseline offset, and increase the resolution of the spectra, but, at the same time, the noise is also amplified. As for the SD pretreatment method, Figure 7e shows that the resolution of the spectra can be increased more obviously, but the noises are also amplified. In addition, the pretreatment combination method is also considered in this study, e.g., SG smoothing combined with the SD method; Figure 7f shows that the noises were not only greatly reduced but also that the spectra bias and baseline offset were reduced.

### 3.4. Spectral Features Extraction

**Figure 8:**
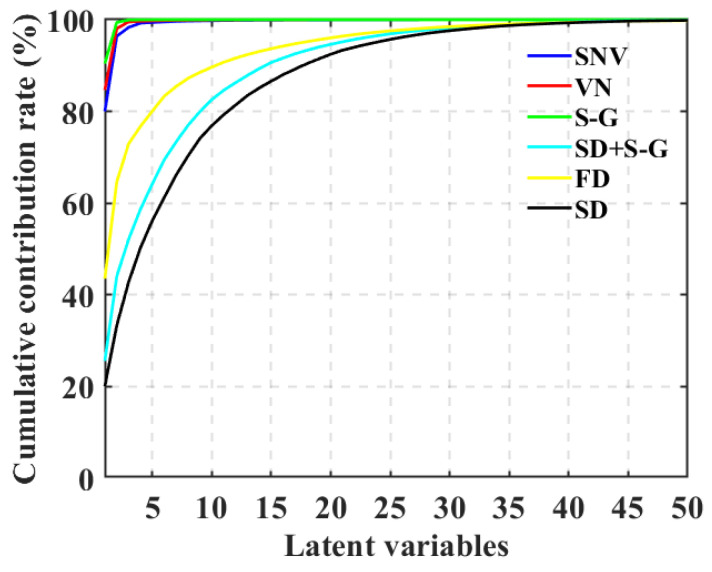
Cumulative contribution rate curves of six different spectra pretreatment methods under different numbers of LVs.

Figure 8 shows the cumulative contribution rates of apples that were Vis-spectra-preprocessed using six pretreatment methods under different LV numbers. The plot of the cumulative contribution rate of the LVs after a dimensionality reduction in the spectra shows that the slope of the curve reflects the changing trend in the contribution rate magnitude of the LVs. Figure 8 shows that the contribution rates of the first LVs for the SNV, VN, and SG smoothing pretreatment methods exceeded 80%, and these are relatively larger than those of the FD, SD, and SD-SG methods, i.e., the first LVs contained most of the spectral information before the dimensionality reduction was performed, and the contribution rate change in the LVs was flat afterward. The changes in the contribution rate magnitude are relatively more moderate.

Figure 8 shows that the cumulative contribution rates of the first 5 LVs for the SNV and VN pretreatment methods basically reached 99–100%, the cumulative contribution rate of the first 3 LVs of the SG pretreatment method also basically reached 99–100%, and the cumulative contribution rates of the first 35 LVs of the FD, SD, and SD-SG pretreatment methods basically reached 99–100%. We found that the first *k* LVs fully represented most information contained in the raw spectra. Based on the PCA mechanism, the maximum amount of dimensionality reduction to model input data can be determined, i.e., the number of LVs corresponding to the desired cumulative contribution rate could be used to represent the original spectral information to the maximum extent possible. In Figure 8, the number of apple spectra LVs pretreated with the SG smoothing, SNV, NV, FD, SD, and SD-SG methods are 3, 5, 5, 35, 35, and 35, respectively. In addition, the score and loading 3D plots of the 3 LVs obtained using the 6 pretreatment methods on the preprocessed spectra are provided in the Supplementary Materials.

### 3.5. SSC Classification Assessments Based on BPNN Models

**Figure 9:**
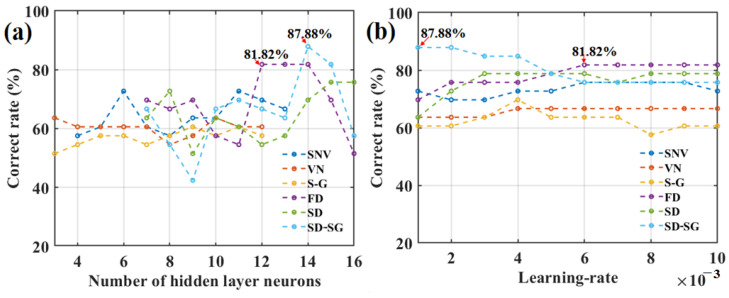
Effects of the number of neurons in the hidden layer (**a**), and the learning rate (**b**) on the correct rate of classifying SSC of the testing-set apples based on six different BPNN models.

Based on the aforementioned six pretreatment methods, six BPNN combination models were established. To obtain satisfactory classification accuracy for apple SSC with each model, several important BPNN parameters were repeatedly adjusted, such as the number of neurons in the hidden layer, the learning rate (*η*), and the training times. The effects of the number of neurons in the hidden layer and the learning rate (*η*) on the correct classification rate regarding apple SSC values (based on six different BPNN models) are shown in Figure 9a,b, respectively.

Figure 9a shows that, under 3000-epoch training times, the correct rates (or accuracies) for classifying SSC levels exceeded 80% for the testing-set apples when the number of neurons in the hidden layer was 14 or 12 for the BPNN model combined with the FD and SD-SG pretreatment methods and the PCA algorithm, i.e., the FD-PCA-BPNN model and the SD-SG-PCA-BPNN model, respectively. However, for the BPNN combined with the other pretreatment methods, the correct SSC classification rates had difficulty reaching 80%. Moreover, Figure 9a shows that the optimal number of neurons in the hidden layer was 14 for the SD-SG-PCA-BPNN model. In addition, Figure 9b shows the correct classification change rate trends for the apple SSC values, the results of which are different compared with the BPNNs combined with other pretreatment methods. For the SD-SG-PCA-BPNN model, the correct rate decreases with an increase in the learning rate. When the learning rate (*η*) is 0.001, the correct rate is the largest (87.88%). For the FD-PCA-BPNN model, when the learning rate (*η*) is 0.006, the correct rate is the largest (81.82%). For the BPNN model combined with other pretreatment methods, their correct classification rates did not reach 80% under all learning rates. Figure 9 preliminarily shows that the classification accuracy of the SD-SG-PCA-BPNN model in determining apple SSC values is better. 

**Figure 10:**
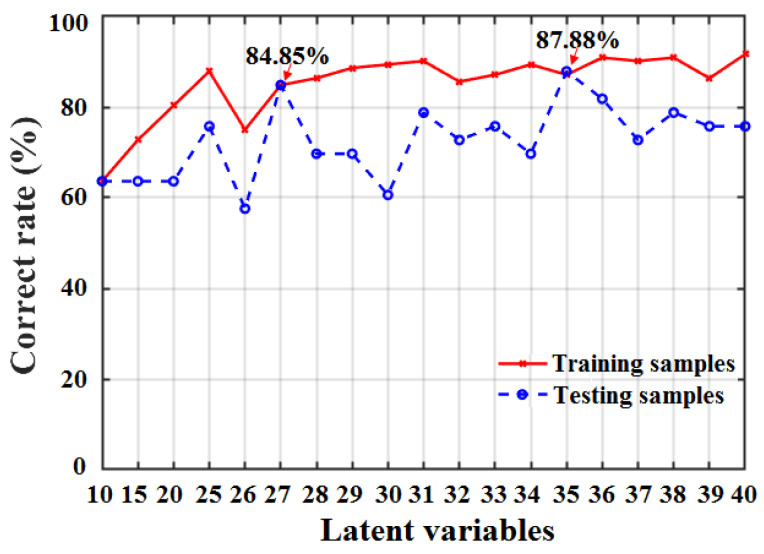
Classification accuracies of apple SSC based on SD-SG-PCA-BPNN model with different LVs.

To further determine the optimal number of LVs for the SD-SG-PCA-BPNN model, the classification accuracies for apple SSC values under different LVs were obtained, as shown in Figure 10.

**Figure 11:**
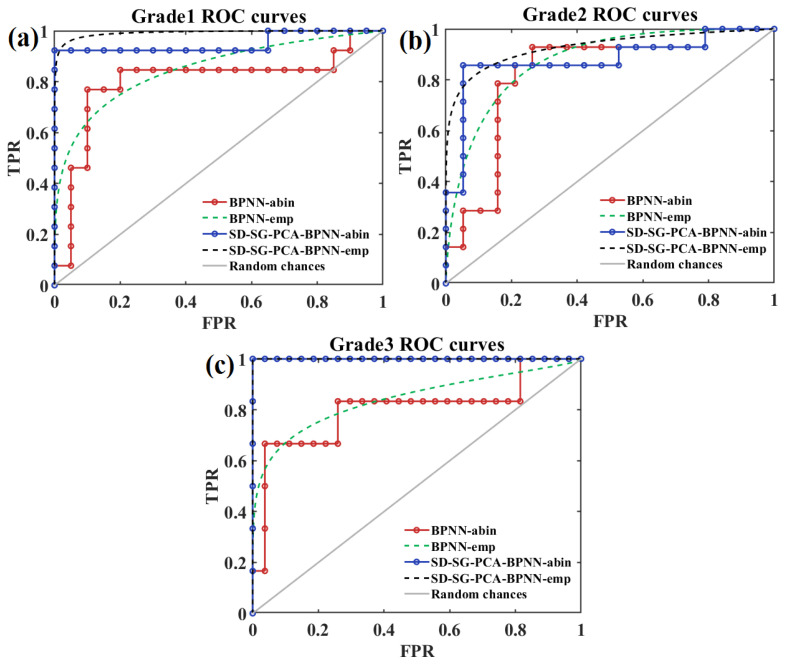
ROC curves for three grades of apples based on SD-SG-PCA-BPNN model. (**a**) grade 1; (**b**) grade 2; (**c**) grade 3.

Figure 10 shows that the classification accuracy in determining apple SSC values is highest when the number of LVs is 35, the number of neurons in the hidden layer is 14, and the learning rate (*η*) is 0.001. To further verify the availability, the receiver operating characteristic (ROC) curves of the three apple grades derived from the SD-SG-PCA-BPNN model (LVs = 35, neurons in the hidden layer = 14, *η* = 0.001) are presented in Figure 11.

Figure 11 shows that the SD-SG-PCA-BPNN model outperforms the BPNN model for all three degrees of classification, and in cases where the false positive rate (FPR) is relatively low, the true positive rate (TPR) is able to quickly approach the upper left region, which means that the SD-SG-PCA-BPNN model has a high hit rate in predicting positive cases and a high accuracy rate in predicting negative cases, i.e., the model has high sensitivity and a low false positive rate. At the same time, its corresponding area under the ROC curve (i.e., AUC) is higher, which also means that the SD-SG-PCA-BPNN model is more capable of distinguishing positive and negative cases; thus, the prediction results are more reliable.

To more intuitively verify the performance of the SD-SG-PCA-BPNN model, the classification results of different BPNN models were compared. Table 2 presents the number of LVs; the optimal number of neurons in the hidden layer; the optimal learning rate (*η*); and the corresponding classification accuracies, recall rates, and *F*_1_ scores for apple SSC values obtained using the BPNN model combined with six pretreatment and PCA methods.

Table 2 shows that the SD-SG-PCA-BPNN model has a higher classification accuracy, recall rate, and F_1_ score regarding apple SSC values than other models. 

### 3.6. SD-SG-PCA-BPNN Model Combined with DLRND Strategy

**Figure 12:**
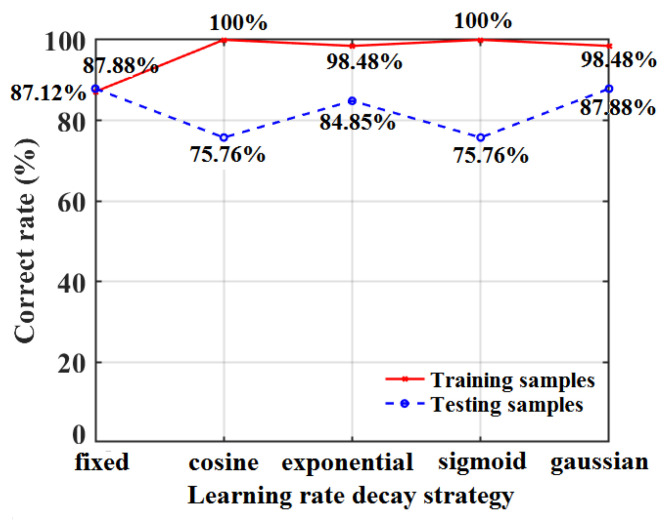
Classification accuracies of apple SSC based on SD-SG-BPNN model with different DLRND strategies.

To solve the constant learning rate problems in the BPNN model, four DLRND strategies (i.e., the exponential strategy, cosine strategy, Gaussian strategy, and Sigmoid strategy; see Equation (4)) were employed and compared in this work. Comparisons were performed between four different DLRND strategies based on the SD-SG-PCA-BPNN model for the training samples and the testing samples, as shown in Figure 12.

Figure 12 shows that the classification accuracies of the cosine DLRND strategy and Sigmoid DLRND strategy reached 100% in the training set using the SD-SG-PCA-BPNN model; however, the classification accuracies in the testing set were lower than those of the fixed learning rate, which may have been caused by model overfitting. The exponential DLRND strategy and Gaussian DLRND strategy have obvious advantages in controlling model overfitting and optimizing the training set’s classification accuracy. For the training samples, the classification accuracy of the SD-SG-PCA-BPNN model, combined with the exponential DLRND strategy and the Gaussian DLRND strategy, is improved to 98.48%, which is better than that of the fixed learning rate. Moreover, the classification accuracy of testing samples using the SD-SG-PCA-BPNN model, combined with the Gaussian DLRND strategy, reaches 87.88%, which is higher than that of the SD-SG-PCA-BPNN model combined with other strategies. Therefore, the Gaussian DLRND strategy is optimal in this work.

**Figure 13:**
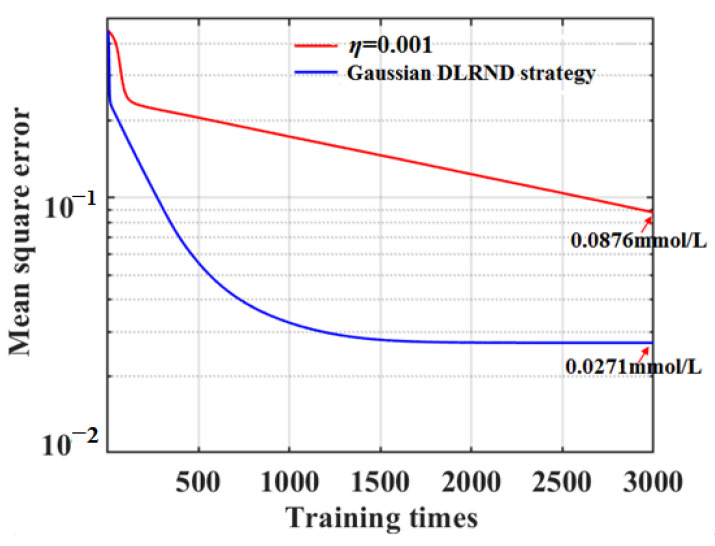
MSE curves based on Gaussian DLRND strategy and fixed learning rate in the training process.

To verify the performance of the SD-SG-PCA-BPNN model combined with the Gaussian DLRND strategy in classifying apple SSCs, its MSE curve was compared with that of the fixed learning rate, as shown in Figure 13.

Figure 13 shows that the Gaussian DLRND strategy is more suitable for cases where the objective function is less complex compared with the fixed learning rate, and another advantage is reflected in the convergent speed improvement. In the model’s training process, the DLRND strategy not only makes convergence faster for the training-set samples but also has a smaller training MSE, which is an obvious improvement in the classification accuracy and convergent speed of the model. However, this does not help much in cases where numerous saddle points and local minima jump out; therefore, it is not effective in improving classification accuracy for the testing-set samples. The training MSE curve of the Gaussian DLRND strategy shows that the training MSE remains basically the same after training times of 1800 epochs; thus, training times of 1800 epochs can achieve a satisfactory classification accuracy for apple SSC values. 

### 3.7. SSC Classification Based on PSO-Optimized SD-SG-PCA-BPNN Algorithm

**Figure 14:**
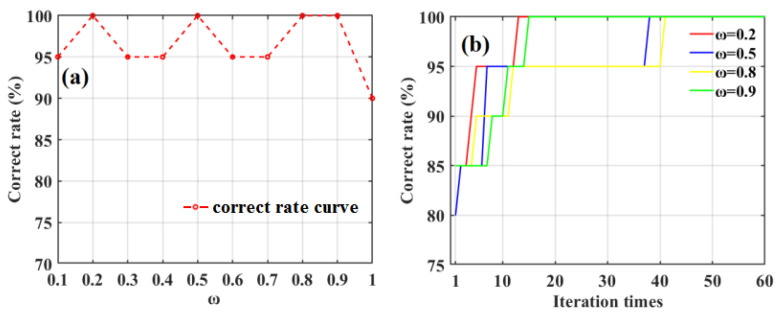
The effect of inertia weight (*ω*) on the correct rate of classifying apple SSC based on SD-SG-PCA-PSO-BPNN model combined with Gaussian DLRND strategy (**a**), and the correct rates of different inertia weights at the PSO iteration evolutionary process (**b**).

To further improve the qualitative accuracy of the SD-SG-PCA-BPNN model in classifying apple SSC values, a PSO algorithm was employed to optimize the parameters of the BPNN model. A PSO algorithm’s performance is usually determined using three main factors, i.e., the inertia weight factor (*ω*), the acceleration factor (*c*_1_), and the acceleration factor (*c*_2_). To obtain *ω*, *c*_1_ and *c*_2_ were both set to two [43]; then, the effect of *ω* on the correct apple SSC classification rate using the SD-SG-PCA-PSO-BPNN model combined with the Gaussian DLRND strategy was investigated, as shown in Figure 14a.

Figure 14a shows that the correct classification rates for the four different inertia-weight values reached 100%, i.e., *ω* = 0.2, 0.5, 0.8, and 0.9. To choose the optimal inertia weight, we obtained the fitness values of four different inertia weights during the PSO evolutionary iteration process, as shown in Figure 14b. Figure 14b shows that, with the increase in iteration times, the correct apple SSC classification rate using the SD-SG-PCA-PSO-BPNN model combined with the Gaussian DLRND strategy gradually increases. Moreover, compared with the correct rate change in other inertia weights, for an inertia weight of 0.2, the classification accuracy can reach 100% when the iteration time is 12 epochs, which is less than those of the other inertia weights. Therefore, the optimal inertia weight (*ω*) is 0.2 in this work. 

Then, *c*_1_ and *c*_2_ should be optimally determined. If *c*_1_ is too small, the algorithm will lose population diversity and easily fall into the local optimum; if so, it cannot jump out. If *c*_2_ is too small, social sharing in the algorithm with no information leads to slow convergence in the algorithm.

**Figure 15:**
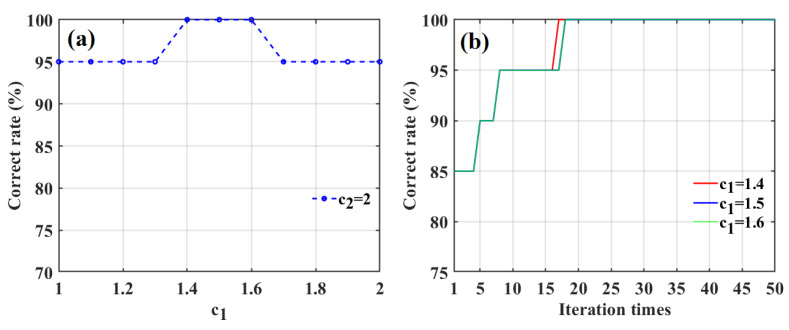
The effect of the acceleration factor *c*_1_ on the classification accuracy of apple SSC based on SD-SG-PSO-BPNN model combined with Gaussian DLRND strategy (**a**), and the correct rates of three different values of the acceleration factor *c*_1_ at the PSO iteration evolutionary process (**b**).

To obtain the optimal values of *c*_1_ and *c*_2_, their effects on classification accuracies for apple sweetness were investigated. Based on past experiences [43], the value of *c*_2_ was initially set to two; the classification accuracy of the SD-SG-PCA-PSO-BPNN model combined with the Gaussian DLRND strategy in determining apple sweetness was obtained using different *c*_1_ values, ranging from 1 to 2 with intervals of 0.1, as shown in Figure 15a.

Figure 15a shows that the correct classification rates for apple SSC values using the SD-SG-PCA-PSO-BPNN model combined with the Gaussian DLRND strategy are the largest (100%) when *c*_1_ is 1.4, 1.5, or 1.6. To determine the optimal acceleration factor, we investigated the effect of iteration times on correct classification rates for apple SSC values with respect to three different values of *c*_1_, as shown in Figure 15b. Figure 15b shows that the iteration times correspond to a 100% correct rate when *c*_1_ is 1.4, which is less than those of the other two values. Therefore, the optimal value of *c*_1_ is 1.4.

**Figure 16:**
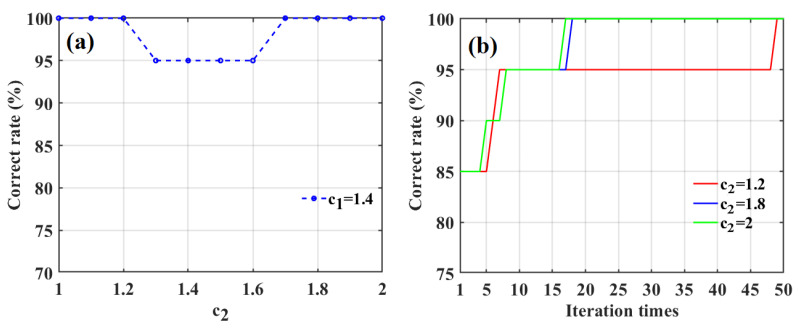
The effect of the acceleration factor *c*_2_ on the classification accuracy of apple SSC based on SD-SG-PCA-PSO-BPNN model combined with Gaussian DLRND strategy (**a**), and the correct rates of three different values of the acceleration factor *c*_2_ at the PSO iteration evolutionary process (**b**).

Then, when *c*_1_ was set to the optimal value, i.e., *c*_1_ = 1.4, we investigated the effect of *c*_2_ on the classification accuracy of the SD-SG-PCA-PSO-BPNN model combined with the Gaussian DLRND strategy in determining apple SCC values, as shown in Figure 16a.

Figure 16a shows that the correct rate is the largest (100%) when *c*_2_ is 1, 1.1, 1.2, 1.7, 1.8, 1.9, or 2. To determine the optimal value of *c*_2_, we investigated the effect of iteration times on the correct classification rates for apple SSC using three different values of *c*_2_ (*c*_2_ = 1.2, 1.8, and 2), as shown in Figure 16b. Figure 16b shows that the iteration times correspond to a correct rate of 100% when *c*_2_ is 2, which is less than those of the other two values. Therefore, the optimal value of *c*_2_ is 2.

**Figure 17:**
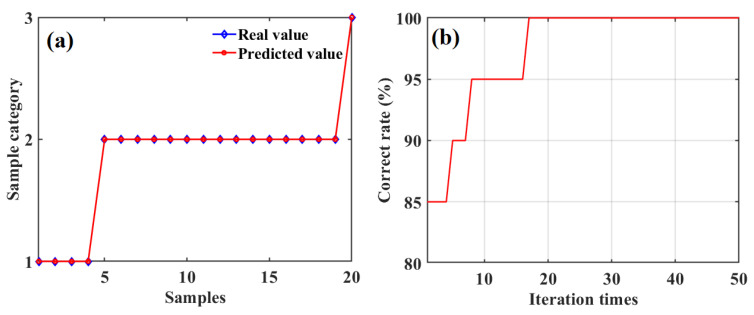
The predicted SSC grades of 20 testing-set apples based on SD-SG-PCA-PSO-BPNN model combined with Gaussian DLRND strategy and three optimal parameters (**a**), and the correct rates of classifying SSC for testing-set apples during the iteration evolutionary process (**b**).

Under the optimal values of the PSO parameters, i.e., *ω* = 0.2, *c*_1_ = 1.4, and *c*_2_ = 2, we obtained the predicted sweetness categories for the testing-set apples using the SD-SG-PCA-PSO-BPNN model combined with Gaussian DLRND strategy, as shown in Figure 17a. The correct classification rates for the testing-set apples’ sweetness during the evolutionary iteration process are shown in Figure 17b. 

Figure 17a,b show that the classification accuracy using the SD-SG-PCA-PSO-BPNN model combined with the Gaussian DLRND strategy in determining SSC grades for the testing-set apples can reach 100% when the iteration times is 17.

### 3.8. Quantitative Assessment of Apple SSC

**Figure 18:**
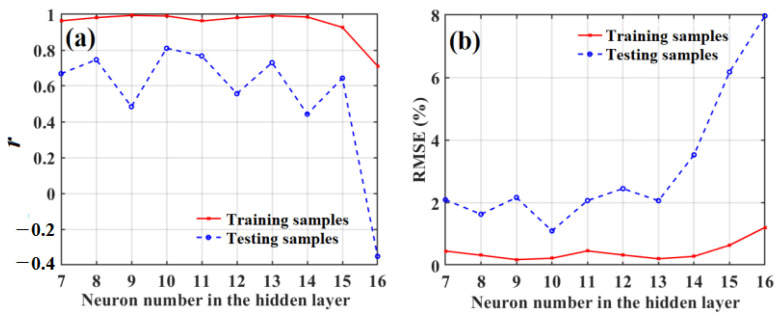
The effect of neuron number in hidden layer on the correlation coefficients (**a**) and RMSE (**b**) of quantitative prediction of apple SSC.

Based on the combined Gaussian DLRND strategy and SD-SG-PCA method in the above-mentioned qualitative analysis of apple SSC values, we then studied the quantitative analysis of apple SSC values using the SD-SG-PCA-PSO-BPNN model combined with the Gaussian DLRND strategy. The data-augmented training-set samples were used to establish the quantitative model, and the raw testing samples were used as the testing set for the quantitative models used to predict SSC values. To find the optimal neuron numbers for the hidden layer of the BPNN in the quantitative model under this condition, we investigated what effect the number of neurons in the hidden layer had on the correlation coefficient (*r*) and root-mean-square error (RMSE) in quantitatively predicting apple SSC values, as shown in Figure 18a,b, respectively.

Figure 18a,b show that the results are unsatisfactory for the testing samples when there are nine neurons in the hidden layer, even though the correlation coefficient (*r*) between the predicted SSC value and the actual SSC value of the training samples is highest and the RMSE value is lowest, i.e., RMSE = 0.2337 °Brix and *r* = 0.99087, and RMSE = 2.1722 °Brix and *r =* 0.48445 for the testing samples. However, when there are 10 neurons in the hidden layer in the training samples, although *r* is slightly lower, and the RMSE is slightly larger than that of the hidden layer with 9 neurons, the correlation coefficient between the predicted SSC and the actual SSC is highest for the testing samples, and the RMSE value is the lowest, i.e., RMSE = 1.1067 °Brix and *r* = 0.81056. Here, the predicted results for the testing samples were used as criteria for determining the optimal number of neurons in the hidden layer of the BPNN in quantitatively predicting apple SSC values. Therefore, the optimal neuron number, in this case, is 10.

**Figure 19:**
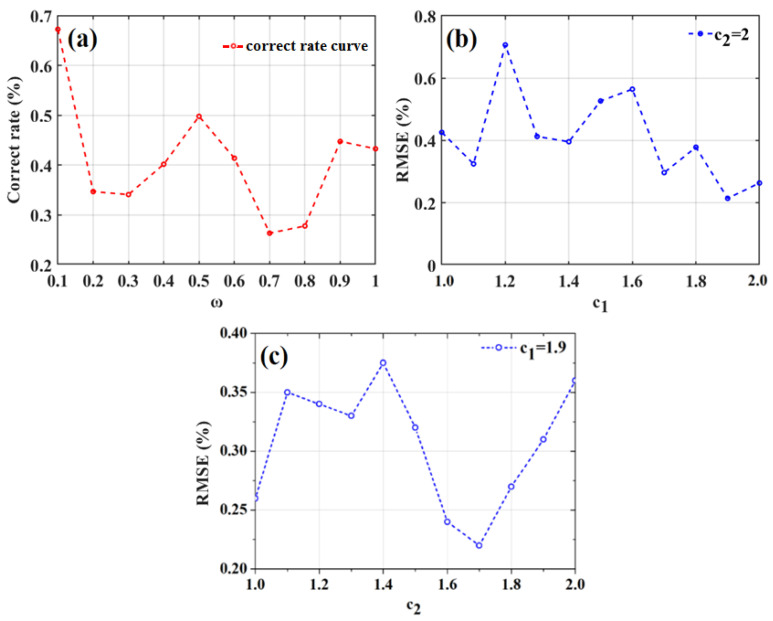
Effects of inertia weight *ω* (**a**), acceleration factor *c*_1_ (**b**) and *c*_2_ (**c**) on the RMSE of quantitative prediction of apple sweetness were investigated based on SD-SG-PCA-PSO-BPNN model combined with Gaussian DLRND strategy.

Then, we investigated the effects of *ω*, *c*_1_, and *c*_2_ on the RMSE in quantitatively predicting apple SSC using the SD-SG-PCA-PSO-BPNN model combined with the Gaussian DLRND strategy, as shown in Figure 19a–c.

Figure 19a–c shows that the RMSE values are smaller when *ω* is 0.7, *c*_1_ is 1.9, and *c*_2_ is 1.7 in quantitatively predicting apple SSC values using the SD-SG-PCA-PSO-BPNN model combined with the Gaussian DLRND strategy. Therefore, the optimal values of *ω*, *c*_1_, and *c*_2_ are 0.7, 1.9, and 1.7, respectively.

**Figure 20:**
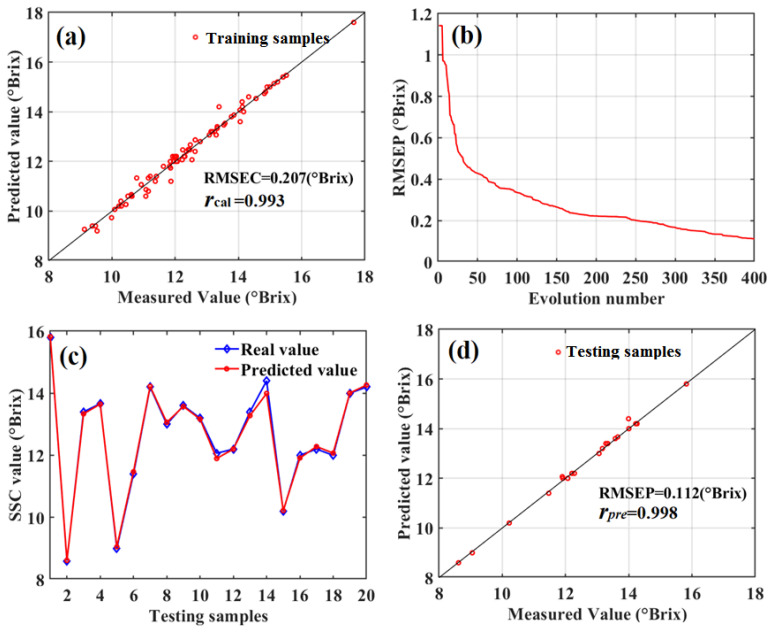
Quantitative predicted SSC for training-set and testing-set apples. (**a**) predicted results of training-set apples; (**b**) the SSC RMSEP curve of testing-set apples under the different evolution numbers of SD-SG-PCA-PSO-BPNN model combined with Gaussian DLRND strategy; (**c**) the predicted SSC and the real SSC of test set apples; (**d**) predicted results of testing-set apples.

Based on the above optimal parameters of the SD-SG-PCA-PSO-BPNN model combined with the Gaussian DLRND strategy, the quantitatively predicted SSC values of 80 training-set apples were obtained and compared with real SSC values, as shown in Figure 20a.

Figure 20a shows the predicted SSC results of the training-set apples have good correlations because the correlation coefficient (*r*) between the predicted SSC values and the real SSC values is 0.993, and the root-square-mean error of calibration (RMSEC) is 0.207 °Brix. Thus, using the SD-SG-PCA-PSO-BPNN model combined with the Gaussian DLRND strategy, the SSC values of 20 testing-set apples were quantitatively predicted. The root-square-mean error of prediction (RMSEP) was used to evaluate the quantitative prediction. The RMSEP curve was obtained using a different evolution number for the SD-SG-PCA-PSO-BPNN model combined with the Gaussian DLRND strategy, as shown in Figure 20b. At an evolution number of 400, the predicted SSC values of the 20 testing-set apples were obtained, as shown in Figure 20c,d, along with the real SSC values. Figure 20c,d show that the quantitative prediction of the testing-set apples’ SSC values was excellent. The RMSEP and correlation coefficient (*r*) of the testing-set apples were 0.112 °Brix and 0.998, respectively. 

## 4. Discussion

### 4.1. Quality Assessments of Fruits Based on Spectroscopy Technique

Table 3 shows different studies on the qualitative and quantitative assessments of fruits and their evaluation results. 

Table 3 shows that most studies on qualitatively classifying and quantitatively detecting fruit quality have focused on NIRS, Vis/NIRS, and Vis/NIR hyperspectral imaging. However, there are relatively few studies on qualitative and quantitative assessments of fruits using only Vis spectroscopy. As with NIRS, Vis spectroscopy also has the advantage of the fingerprint effect, which can be used to qualitatively and quantitatively assess the quality of fruits. More importantly, Vis spectroscopy has a unique feature, i.e., inexpensive instruments, which can ensure a higher performance-to-cost ratio in qualitatively and quantitatively assessing fruit quality, even promoting the extension of fruit sorting and grading scales. Therefore, this work explored a kind of cost-effective and high-accuracy optical assessment of apple quality; Vis spectroscopy, combined with ANN models and optimization methods, was employed to qualitatively and quantitatively assess the quality of apples based on SSC values. Table 3 shows that, with the synthetic model proposed in this work, i.e., the SD-SG-PCA-PSO-BPNN model combined with the Gaussian DLRND strategy, the qualitative classification accuracy for apple SSC values can reach 100%, and the RMSEP and correlation coefficient (r) can reach 0.112 °Brix and 0.998, respectively, which are superior to the results of other methods.

### 4.2. Comparison of Qualitative Assessments between Different Algorithms

**Figure 21:**
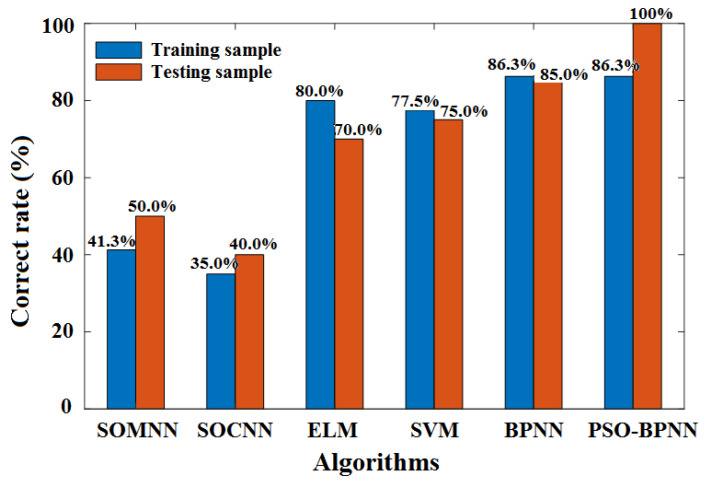
Comparison between classification accuracies of apples’ SSC based on different algorithms.

To evaluate the qualitative classification performance of the synthetic models proposed in this work, an accuracy analysis for the augmented training-set and the raw testing set was performed. Based on the Vis spectra of apples pretreated using the SD-SG and PCA methods, the correct classification rates for 131 groups of training-set apples and 20 groups of raw-testing-set apples for several different algorithms were compared, such as the self-organizing mapping neural network (SOMNN) [50,51], the self-organizing competitive neural network (SOCNN) [52,53], the extreme learning machine (ELM) [54], support vector machines (SVM) [55,56], the BPNN model combined with the Gaussian DLRND strategy, and the PSO-BPNN model combined with the Gaussian DLRND strategy, as shown in Figure 21.

Figure 21 shows that, under the same spectra pretreatment methods, i.e., SD-SG and PCA, the classification accuracies of the SOMNN and SOCNN algorithms for the training apple samples and the testing apple samples are all below 60%, which indicates the classification performance of SOMNN and SOCNN in determining apple SSC values is not satisfactory because of the high similarity between each sample grade. The classification accuracies of the ELM and SVM algorithms for the training and testing samples were higher than 70% but less than 80%. Although the accuracies of ELM and SVM are higher than those of SOMNN and SOCNN, their performance is still not satisfactory. The classification accuracies of the SD-SG-PCA-BPNN algorithm for the training apple samples and testing apple samples are 86.3% and 85%, respectively. However, for the SD-SG-PCA-PSO-BPNN model combined with the Gaussian DLRND strategy algorithm, although the classification accuracy for the training apple samples was still 86.3%, the classification accuracy for the testing apple samples reached 100%. Therefore, the qualitative classification performance of the SD-SG-PCA-PSO-BPNN model combined with the Gaussian DLRND strategy algorithm is the best for determining apple SSC values.

### 4.3. Comparison of Quantitative Assessments between Different Algorithms

To further verify the availability and performance of quantitative predictions for apple SSC values based on the synthetic model proposed in this work, i.e., the SD-SG-PCA-PSO-BPNN model combined with the Gaussian DLRND strategy, three quantitative prediction models were compared, i.e., SD-SG-PCA combined with partial least squares regression (i.e., SD-SG-PCA-PLSR), SD-SG-PCA-BPNN, and the SD-SG-PCA-PSO-BPNN model combined with the Gaussian DLRND strategy. Using the same pretreatment method and number of LVs, i.e., the SD-SG pretreatment algorithm and 35 LVs, the correlation coefficient of the training set (*r*_cal_) and the correlation coefficient of the testing set (*r*_pre_), as well as the RMSEC and RMSEP values, were all obtained, as shown in Table 4.

Table 4 shows that, under the same pretreatment method, the SD-SG-PCA combination algorithm could pretreat the raw Vis spectra of apples; the *r*_cal_ and RMSEC values were 0.824 and 0.981 °Brix for the training-set apples and the *r*_pre_ and RMSEP values were 0.758 and 1.417 °Brix for the testing-set apples, as per the SD-SG-PCA-PLSR model with 35 LVs. These results are better than those of the SD-SG-PCA-PLSR model with fewer LVs (e.g., 5 and 15 LVs). For the SD-SG-PCA-BPNN model, when using the SD-SG and 35-LV pretreatment method and Gaussian DLRND strategy, *r*_cal_ and RMSEC were 0.991 and 0.224 °Brix for the training-set apples, and *r*_pre_ and RMSEP were 0.814 and 1.107 °Brix for the testing-set apples. For the SD-SG-PCA-PSO-BPNN model combined with the Gaussian DLRND strategy, *r*_cal_ and RMSEC were 0.993 and 0.207 °Brix for the training-set apples, and *r*_pre_ and RMSEP were 0.998 and 0.112 °Brix for the testing-set apples. At the same time, the root-mean-square error of cross-validation (RMSECV) relating to apple SSC values obtained using three different algorithms was obtained using the leave-one-out method; as such, the RMSECV of the SD-SG-PCA-PSO-BPNN model combined with the Gaussian DLRND strategy in determining apple SSC values is the smallest. Moreover, the RMSEP of the SD-SG-PCA-PSO-BPNN model combined with the Gaussian DLRND strategy is less than the RMSECV. Therefore, the synthetic method proposed in this work, i.e., the SD-SG-PCA-PSO-BPNN model combined with the Gaussian DLRND strategy, has better performance than the two others, and it has significant value in qualitatively classifying and quantitatively predicting apple SSC values. 

## 5. Conclusions

This work explored a cost-effective optical assessment of apple quality, i.e., Vis spectroscopy combined with an ANN model, which was used to qualitatively classify and quantitatively predict the SSC level of apples. To ensure the high assessment accuracy of apple SSC values, the raw Vis spectra of the apples were preprocessed using six pretreatment methods and PCA; the optimal pretreatment methods were determined using the BPNN model. For the SD-SG-PCA-BPNN model, the apple SSC classification accuracy was 87.88%; the recall rate was 90.29%; the *F*_1_ score was 0.8755 when the number of LVs was 35; the number of neurons in the hidden layer was 14; and the learning rate (*η*) was 0.001. These results are better than those of the other pretreatment methods. Thus, to improve the qualitative classification accuracy and convergent speed, four DLRND strategies were used to train the BPNN and then compared. The faster convergent speed and higher classification accuracy for apple SSC values were obtained using the SD-SG-PCA-BPNN model combined with the Gaussian DLRND strategy; the classification accuracy of the training set and the testing set reached 98.48% and 87.88%, respectively. Furthermore, the PSO algorithm was used to optimize the SD-SG-PCA-BPNN model combined with the Gaussian DNLRD strategy. The classification accuracy reached 100%. In addition, the apple SSC quantitative assessment was performed using the SD-SG-PCA-PSO-BPNN model combined with the Gaussian DLRND strategy. Under the optimal parameters, the RMSEC and correlation coefficient (*r*_cal_) for the apple SSC values in the training set were 0.207 °Brix and 0.993, respectively; the RMSEP and correlation coefficient (*r*_pre_) of the apple SSC values for the testing set were 0.112 °Brix and 0.998, respectively; and the leave-one-out RMSECV was 0.2693%. These results are all better than those of the PLSR and BPNN models. At the same time, the classification accuracy and RMSEP regarding apple SSC in our work were also better than those of other studies. The results show that qualitatively and quantitatively assessing apple quality can be significantly achieved via Vis spectroscopy and an SD-SG-PCA-PSO-BPNN model combined with a Gaussian DLRND strategy. Furthermore, this indicates that the synthetic method can be extended to qualitative and quantitative assessments of other fruits.

## Figures and Tables

**Table 1:** The statistical analysis results for SSC values of apple samples.

Grade	SSC Range (°Brix)	Minimum (°Brix)	Maximum (°Brix)	Mean (°Brix)	Variance (°Brix)	SE of Mean * (°Brix)	MAD ** (°Brix)	CV *** (%)
1	14–17.99	14.2	17.6	14.83	0.668	0.17423	0.5909	0.0551
2	10–13.99	10.2	13.867	12.06	1.136	0.12649	0.8512	0.0884
3	8–9.99	8.6	9.733	9.23	0.127	0.1348	0.2531	0.0386

SE of Mean *: standard error of the mean; MAD **: mean absolute deviation; CV ***: coefficient of variation.

**Table 2:** Comparison of classification accuracies, recall rates and *F*_1_ scores of apple SSC based on BPNN models combined with six kinds of pretreatment methods.

No.	Models	LVs	Neuron Number in Hidden Layer	Learning Rate	Classification Accuracy/%	Recall Rate/%	*F*_1_ Score/%
1	SNV-BPNN	8	6	0.006	75.76	74.05	0.7675
2	VN-BPNN	5	10	0.004	66.67	63.55	0.6440
3	SG-BPNN	3	11	0.004	69.70	66.12	0.6786
4	FD-BPNN	35	12	0.006	81.82	79.18	0.8003
5	SD-BPNN	35	16	0.003	78.79	80.04	0.7705
6	SD-SG-BPNN	5	14	0.001	60.61	49.27	0.6451
		10	14	0.001	63.64	52.01	0.7387
		35	14	0.001	87.88	90.29	0.8755
7	BPNN	81	13	0.001	78.79	76.43	0.7758

**Table 3:** Different qualitative and quantitative studies of fruits quality and their evaluation results.

Reference	Object	Spectroscopy	Model	Evaluation Indicators
Ref. [4]	Apple	Vis/NIRS hyperspectral imaging	PLSR	*R*^2^ = 0.802, RMSE = ±0.674 °Brix (SSC)
Ref. [5]	Apple	Vis/NIRS hyperspectral imaging	PLS-DA	Accuracy of variety: 99.4%
Ref. [6]	Apple	NIRS	BP, GRNN^1^	MAPE^2^ = 5.41% (acidity); MAPE = 13.95% (sweetness)
Ref. [7]	Apple	NIRS	PCA-DA	Accuracy of origin traceability: 93.6% (high-elevation), 77.9% (low-elevation)
Ref. [8]	Apple	Vis/SW-NIRS	SFS^3^, LDA	Accuracy range: 87.3–97.6% (firmness); 77.1–92.3% (SSC)
Ref. [9]	Apple	NIRS	PLS-DA	Classification accuracy = 96%
Ref. [10]	Apple	NIRS	DA	Classification accuracy = 91.3% (sweetness)
Ref. [11]	Apple	NIRS	SPA-LS-SVM	Classification accuracy = 90.11% (ripeness)
Ref. [12]	Shatian pomelo	Vis/NIRS	SG-MSC-GA-PCA-CNN-PLSR	*R*^2^ = 0.72, RMSE = 0.49 °Brix (SSC) *R*^2^ = 0.55, RMSE = 0.10% (acidity)
Ref. [13]	Wax apple	Vis/SW-NIRS	PLSR	*R*_p_^2^ = 0.87, RMSEP = 0.66 °Brix(SSC) *R*_p_^2^ = 0.80, RMSEP = 1.16 N/cm^2^ (firmness)
Ref. [14]	Apple	Vis/NIRS	MCARS^4^ and SPA-PLS	*r* = 0.946, RMSE = 0.527 °Brix for prediction set (SSC)
Ref. [15]	Golden apple	Vis/NIRS	LDA, QDA and SVM	Classification accuracy range: 75–81% (bitter pit)
Ref. [16]	Nectarine, peach, apricot and Japanese plums	Vis/NIRS	SD-PLSR	Classification accuracy: >75% (SSC, DMC and flesh firmness)
Ref. [45]	Maize	Vis/NIRS hyperspectral imaging	LDA and ANN	Classification accuracy: 95% (LDA), 85% (ANN)
Ref. [46]	Winter wheat	Vis/NIRS	SNV-SG-PLS and ANN	PLS: *r* = 0.92 and RMSE = 0.9131; ANN: *r* = 0.97 and RMSE = 0.7305 (LCC^5^)
Ref. [47]	Tomato	Vis/NIRS	PLS-DA	Accuracy of pesticide residue: 91.66% (prediction sets); SECV^6^ = 4.2767
Ref. [48]	Potato	Vis/NIRS	PLS, ANN	Accuracy: PLS: 89% (SSC) and 93% (pH); ANN: 85% (SSC) and 90% (pH)
Ref. [49]	Potato	NIRS	PCA, PLS	*R*^2^ > 0.80
Ours	Apple	Vis spectroscopy	SD-SG-PCA-PSO-BPNN	Classification accuracy: 100%; RMSEP = 0.112 °Brix; *r* = 0.998 (SSC)

GRNN^1^: generalized regression neural network; MAPE^2^: mean absolute percentage error; SFS^3^: sequential forward selection; MCARS^4^: modified competitive adaptive reweighted sampling; LCC^5^: leaf chlorophyll content; SECV^6^: standard error of cross validation.

**Table 4:** The comparison of quantitative prediction of apples’ SSC based on different models.

Model	Pretreatment	LVs	Training Set		Testing Set		Leave-One-Out RMSECV (°Brix)
			*r*_cal_	RMSEC (°Brix)	*r*_pre_	RMSEP (°Brix)	
PLSR	SD-SG	5	0.388	1.597	0.527	1.540	1.3782
		10	0.693	1.248	0.537	1.592	1.2117
		35	0.824	0.981	0.758	1.417	1.3254
BPNN		35	0.991	0.224	0.814	1.107	0.8215
PSO-BPNN		35	0.993	0.207	0.998	0.112	0.2693

## Data Availability

All related data and methods are presented in this paper. Additional inquiries should be addressed to the corresponding author.

## Supplementary Materials

The following supporting information can be downloaded at: https://www.mdpi.com/article/10.3390/foods12101991/s1, Figure S1: The score and loading plots for six different pretreatment methods with PCA of 3 Lvs.
