# Supplementary material for: Qualitative and Quantitative Assessments of Apple Quality Using Vis Spectroscopy Combined with Improved Particle-Swarm-Optimized Neural Networks
Source: Foods. 2023 May 15;12(10):1991. doi: 10.3390/foods12101991 (PMC10217276; doi:10.3390/foods12101991)
Supplement: Supplementary file 1 [file foods-12-01991-s001.zip › foods-2355456-supplementary.pdf]

Supplementary materials:

The score and loading plots for six different pretreatment methods with PCA of 3 LVs are shown in Figure S1.

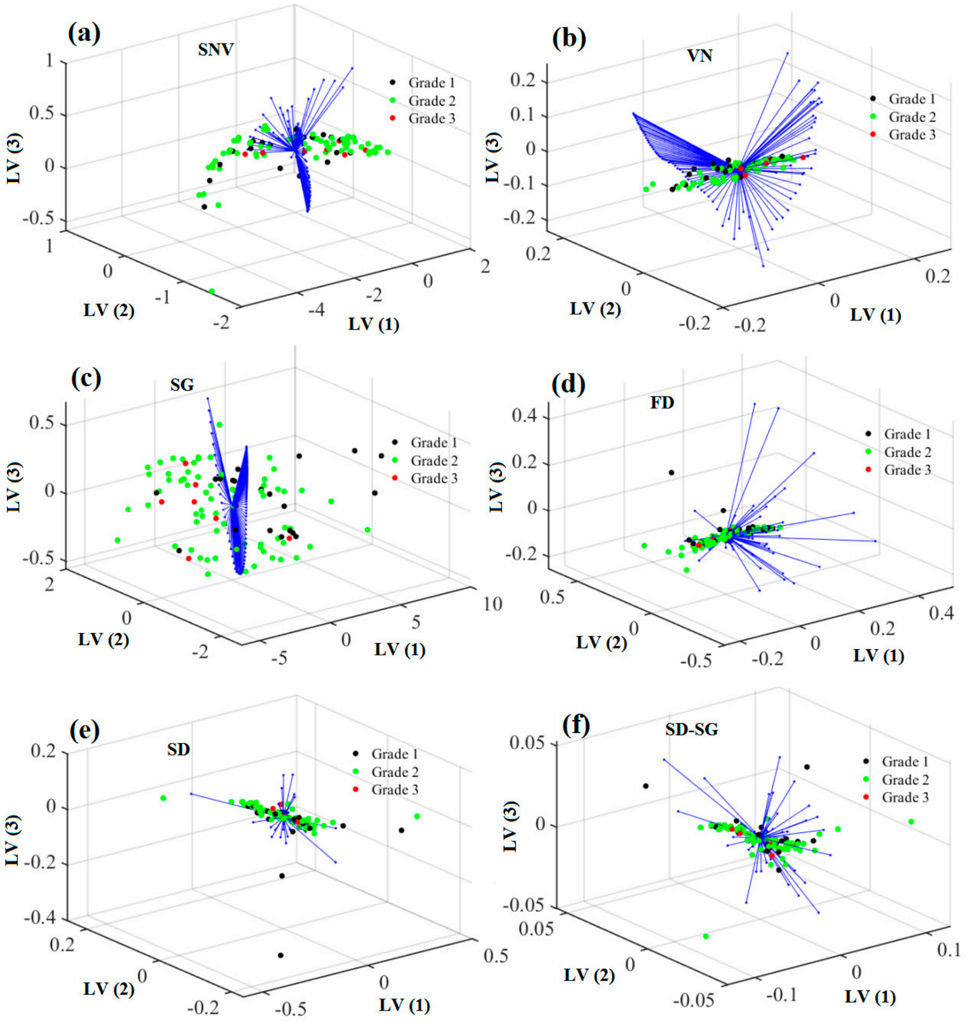

Figure S1. The score and loading plots for six different pretreatment methods with PCA of 3 Lvs.

Figure S1a–c show that the differentiation and correlation between samples are not significant for the spectra preprocessed by the SNV, VN and SG pretreatment methods. However, from the Figure S1d–f, it can be seen that the differentiation and correlation between samples for the spectra preprocessed by the FD, SD and SD-SG pretreatment methods are better than those of the SNV, VN and SG pretreatment methods.
